# Identification and Validation of an Immune-Associated RNA-Binding Proteins Signature to Predict Clinical Outcomes and Therapeutic Responses in Glioma Patients

**DOI:** 10.3390/cancers13071730

**Published:** 2021-04-06

**Authors:** Ruotong Tian, Yimin Li, Qian Liu, Minfeng Shu

**Affiliations:** 1 Department of Pharmacology, School of Basic Medical Sciences, Shanghai Medical College, Fudan University, No.131 Dong’an Road, Xuhui District, Shanghai 200032, China; 20211010075@fudan.edu.cn (R.T.); 18211010067@fudan.edu.cn (Q.L.); 2 Department of Pathology, Fudan University Shanghai Cancer Center, No.270 Dong’an Road, Xuhui District, Shanghai 200032, China; 176501028@csu.edu.cn

**Keywords:** glioma, RNA-binding protein, immune microenvironment, tumor-infiltrating immune cells, risk score, prognostic model, immunotherapy, chemotherapy

## Abstract

**Simple Summary:**

Both of tumor-infiltrating immune cells and the RNA-binding proteins (RBPs) that are able to mediate immune infiltration contribute to the prognosis of patients with glioma. However, immune-associated RBPs in glioma remain unexplored. In this study, we developed a method to identify RBPs associated with immune infiltration in glioma and 216 RBPs were defined as immune-associated RBPs. Among them, eight RBPs were selected to construct a risk signature that proved to be a novel and independent prognostic factor. Higher risk scores meant worse overall survival and higher expression of human leukocyte antigen and immune checkpoints. Additionally, analyses of pathway enrichment, somatic mutation, copy number variations, and immuno-/chemotherapeutic response prediction were performed to evaluate the differences between high- and low-risk groups. Generally, we demonstrated an eight immune-associated RBPs prognostic signature that was valuable in predicting the survival of glioma patients and directing immunotherapy and chemotherapy.

**Abstract:**

The prognosis of patients with glioma is largely related to both the tumor-infiltrating immune cells and the expression of RNA-binding proteins (RBPs) that are able to regulate various pro-inflammatory and oncogenic mediators. However, immune-associated RBPs in glioma remain unexplored. In this study, we captured patient data from The Cancer Genome Atlas (TCGA) and divided them into two immune subtype groups according to the difference in infiltration of immune cells. After differential expression and co-expression analysis, we identified 216 RBPs defined as immune-associated RBPs. After narrowing down processes, eight RBPs were selected out to construct a risk signature that proven to be a novel and independent prognostic factor. The patients were divided into high- and low-risk groups on the basis of risk score. Higher risk scores meant worse overall survival and higher expression of human leukocyte antigen and immune checkpoints such as PD1 and CTLA4. In addition, analyses of pathway enrichment, somatic mutation, copy number variations and immuno-/chemotherapeutic response prediction were performed in high- and low-risk groups and compared with each other. For the first time, we demonstrated a novel signature composed of eight immune-associated RBPs that was valuable in predicting the survival of glioma patients and directing immunotherapy and chemotherapy.

## 1. Introduction

Glioma is regarded as the most common and lethal type of primary brain tumor with an extremely poor prognosis, accounting for about 30 percent of all central nervous system (CNS) tumors and 80 percent of all primary malignant brain tumors [1]. According to the World Health Organization (WHO) classification of CNS tumors, malignant adult diffused gliomas are classified into grades II to IV based on histologic features. In the 2016 edition, isocitrate dehydrogenase (IDH) mutation and chromosomal co-deletion 1p/19q are integrated into the traditional glioma classification [2]. But even if gliomas are subdivided into more subtypes based on molecular and histological features, these indicators remain very limited on prognosis assessment and optimization of therapy regimen. Therefore, a novel method to identify the risk of glioma patients and who is most likely to benefit from adjuvant therapy will bring immense value for personalized cancer care.

The unique immunological status in the CNS contributes to the particular glioma tumor microenvironment. A variety of immune cells including brain resident microglia and peripheral immune cells are present in glioma microenvironment [3]. Tumor-derived cytokines and chemokines are able to reprogram those immune cells into tumor-associated phenotypes which therefore have profound effects on progression and therapeutic resistance by inducing inflammatory or anti-inflammatory responses [4]. The multiple therapeutic antibodies that block immune checkpoints, such as cytotoxic programmed cell death protein 1 (PD1, PDCD1) and T lymphocyte associated antigen 4 (CTLA4), showed great effects in treating non-small-cell lung cancer, kidney cancer, and melanoma [5]. Yet the clinical application of checkpoint inhibitors in glioma is up against tremendous challenges because of the “cold phenotype” of glioma characterized by a paucity of T-cell infiltration but robust macrophage infiltration [4,6,7]. Therefore, the role of immune cells and the factors that regulate the infiltration of immune cells need to be studied urgently.

As trans-acting factors, RNA-binding proteins (RBPs) largely contribute to the supervision of gene expression by interacting with target RNAs [8], which participate in the initiation and progression of glioma [9,10,11,12]. Specifically, RBPs regulate almost all aspects of RNA life, including pre-mRNA processing, modification, localization, RNA stability, and translation [13]. Post-transcriptional regulation of inflammatory mRNAs, mediated by a set of RBPs including Tristetraprolin, Roquin and Regnase-1, and RNA methylases, is increasingly understood to rapidly respond to inflammatory mediators and orchestrate the inflammatory response by modulating mRNA pools in both immune and nonimmune cells [14,15]. Considering that RBPs can largely regulate the RNA pool related to inflammation and the infiltration degree as well as specific function of immune cells depend on tumor-derived cytokines and chemokines, whether there are immune-associated RBPs that can be used to accurately evaluate the tumor progression and prognosis of glioma patients has not yet been considered.

In this study, we captured data from The Cancer Genome Atlas (TCGA) and then divided glioma patients into two immune subtype groups based on single sample Gene Set Enrichment Analysis (ssGSEA) score of 24 types of immune cells. Next, differentially expressed RBPs between two immune subtype groups correlated with at least one type of immune cell were defined as immune-associated RBPs for further analysis. Univariate Cox regression analysis, least absolute shrinkage and selection operator (LASSO) regression analysis, and multivariate Cox regression analysis were used to construct a prognostic signature. Glioma patients from TCGA and the Chinese Glioma Genome Atlas (CGGA) were separately classified into low- and high-risk groups in the light of risk scores based on the prognostic signature. Analyses of pathway enrichment, somatic mutation and copy number variations (CNVs), and immuno-/chemotherapeutic response prediction were performed in the two risk groups.

## 2. Materials and Methods

### 2.1. Data Acquisition

The transcriptome data and corresponding clinical parameters were downloaded from TCGA database (https://portal.gdc.cancer.gov Assessed on 19 July 2019) and CGGA database (http://www.cgga.org.cn/ Assessed on 21 December 2020). A total of 1709 glioma patients were included in this analysis (TCGA: 691 patients; CGGA: 1018 patients.). Somatic mutation and CNVs data of glioma patients were also obtained from TCGA database. Somatic mutation data were analyzed using “maftools” package, and significant amplifications or deletions of copy number were detected using GISTIC 2.0. The list of RNA-binding proteins was acquired through a previously published article [16].

### 2.2. Identification of Glioma Immune Subtypes Based on ssGSEA Score

With the aim of exploring the infiltration level of different immune cell populations, a set of marker genes defining various types of immune cell was first obtained from the work of Gabriela Bindea et al. [17]. The ssGSEA algorithm is a rank-based method that defines a score representing the degree of absolute enrichment of a particular gene set in each sample [18]. To calculate ssGSEA scores, we used the GSEA program to obtain the absolute enrichment scores of gene signature of immune cells according to previous studies [19,20,21]. In brief, the infiltration levels of immune cells were quantified by ssGSEA in “Gene Set Variation Analysis (GSVA)” package using default parameters [22]. The “ConsensusClusterPlus” package was used for consensus clustering and distinguishing different immune subtypes based on ssGSEA scores [23]. The consistent matrix (CM) plots were illustrated based on k-value. The empirical cumulative distribution function (CDF) plots revealed the consensus distributions for each k [24]. The purpose of the CDF plot is to find the k at which the distribution reaches an approximate maximum, which indicates a maximum stability and after which divisions are equivalent to random picks rather than true cluster structure [23]. According to the results of K-means clustering, samples of glioma patients in the TCGA database were classified into two immune subtype (Sub) groups: Sub1 group and Sub2 group. The Tumor Purity, ESTIMATE Score, Immune Score, and Stromal Score were analyzed by ESTIMATE algorithm [25]. The CIBERSORT deconvolution algorithm (https://cibersort.stanford.edu/ Assessed on 28 December 2020) was used to verify that the infiltration of immune cells from these two Sub groups was different [26].

### 2.3. Identification of Immune-Associated RBPs in Glioma

Given the differences in immune cell infiltration between Sub1 and Sub2 groups, “limma” package was conducted for seeking differentially expressed RBPs. The thresholds were set as/log_2_ fold change (FC)/ > 1 and adjusted *p* value < 0.05. Correlation analysis was further performed between differential expression RBPs and ssGSEA scores of 24 types of immune cells. An RNA-binding protein whose expression value was correlated with at least one immune cell (|Pearson R| > 0.6 and adjusted *p* value < 0.05) was defined as the immune-associated RBP.

### 2.4. Risk-Based Modeling

First, univariate Cox regression analysis was conducted to evaluate the relevance between the patient’s overall survival and their transcriptome data of immune-associated RBPs in TCGA database with *p* value < 0.001 as the criteria. Then, LASSO regression analysis was performed and 10-round cross-validation was used to prevent overfitting. Finally, multivariate Cox analysis was used to work out the coefficients and construct a prognostic signature. The risk score formula is as follows: risk score = Σ*Coefficient* (RBP*i*) × *Expression* (RBP*i*). According to the median of risk score, glioma patients were divided into low- and high-risk group. The Kaplan–Meier curve was used to assess the differences of overall survival between low- and high-risk group by “survminer” package. The time-dependent receiver operating characteristic (ROC) curve was performed and the area under the ROC curve (AUC) was also calculated by “pROC” package. Univariate and multivariate Cox regression analysis was used to evaluate the independence of prognostic gene signature and other clinical parameters (age, sex, grade, ATRX, status, IDH status, MGMT promoter, and immune subtypes). We combined the clinical parameters with the eight immune-associated RBPs signature to construct a nomogram using “rms” package. The C-index was used to evaluate the discriminative power of the nomogram and draw a calibration chart to evaluate the accuracy of the nomogram.

### 2.5. Pathway Enrichment Analysis

We applied DAVID database (https://david.ncifcrf.gov/ Assessed on 20 January 2021) to complete the Gene ontology (GO) and the Kyoto Encyclopedia of Genes and Genomes (KEGG) pathway enrichment analysis. GO or KEGG pathways with adjusted *p* value < 0.05 were considered statistically significant. GSVA (http://www.bioconductor.org/packages/release/bioc/html/GSVA.html Assessed on 30 January 2021) and Gene set enrichment analysis (GSEA) (http://www.broadinstitute.org/gsea/index.jsp Assessed on 30 January 2021) was performed to detect a significant difference in the set of genes expressed between the low- and high-risk groups [22,27]. The thresholds were as follows: GSVA, the *p* value < 0.05 and the t value > 2; GSEA, *p* value < 0.05 and false discovery rate (FDR) < 0.25.

### 2.6. Immuno-/Chemotherapeutic Response Prediction

As reported in the previous articles [28,29], the subclass mapping was used to predict the clinical response to immune checkpoints inhibitors between the low- and high-risk group in TCGA database [30]. We also used “Prophetic” package to predict the chemotherapy response of each sample based on Genomics of Drug Sensitivity in Cancer (GDSC) (https://www.cancerrxgene.org/ Assessed on 31 January 2021). Nine common chemotherapeutic agents (Bleomycin, Cisplatin, Cyclopamine, Docetaxel, Doxorubicin, Etoposide, Gemcitabine, Paclitaxel, and Vinblastine), small molecule inhibitors targeting EGFR (Erlotinib and Lapatinib) and targeting VEGFR (Pazopanib and Sunitinib) and Metformin were selected and kept the default values for all parameters.

### 2.7. Statistical Analysis

Kaplan–Meier curve and log-rank test were adopted to assess whether there were differences in overall survival between groups. Statistical analyses involved in this research were conducted through R software (version 3.6.3, https://www.r-project.org/ Assessed on 31 January 2021). A χ2 or Fisher’s exact test was performed for categorical data. A Student’s *t*-test or Wilcoxon test was performed for continuous data. For all statistical analyses, *p* value < 0.05 was considered statistically significant.

## 3. Results

### 3.1. Identification of Glioma Immune Subtypes Based on Infiltration of Immune Cells

**Figure 1:**
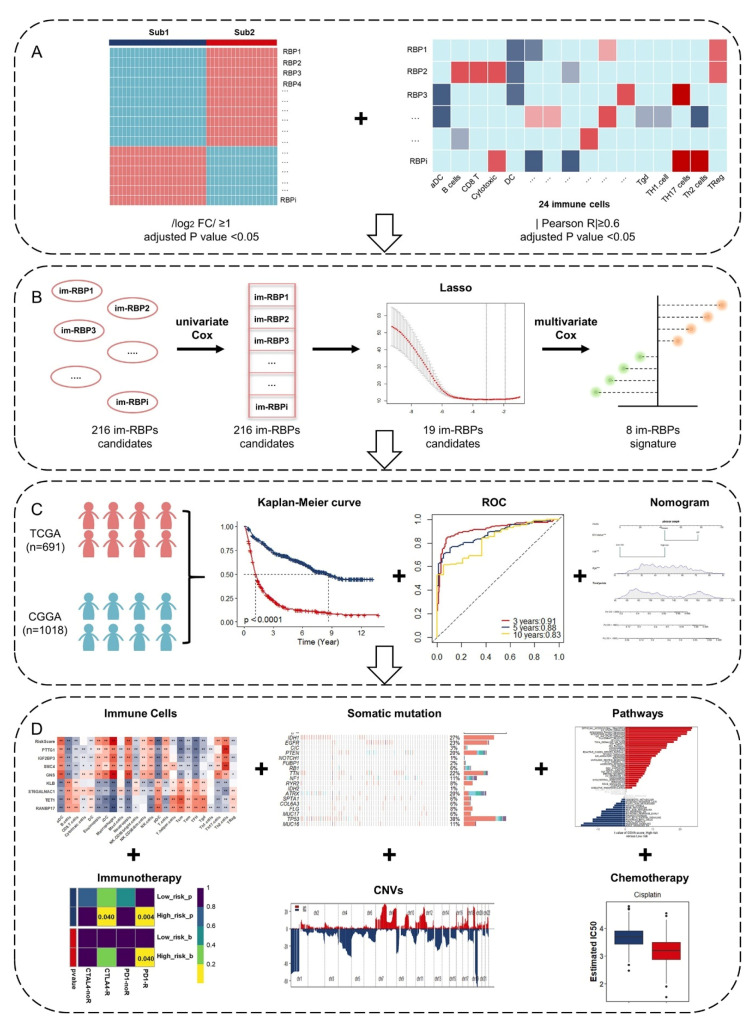
Schematic workflow for analyzing immune-associated RBPs in glioma: (**A**) Two immune subtypes identified by ssGSEA and “ConsensusClusterPlus” package, and the differentially expressed RBPs singled out by “limma” package between two subtype groups. Correlation analysis between differentially expressed RBPs and immune cells. (**B**) A combination of the LASSO and Cox regression analysis to identify a prognostic signature based on immune-associated RBPs (im-RBPs: immune-associated RBPs). (**C**) Identification and assessment of the prognostic signature of immune-associated RBPs for overall survival of glioma patients in TCGA and CGGA. (**D**) Comprehensive analyses of tumor-infiltrating immune cells, responses to immunotherapy, somatic mutations, copy number variations, enriched pathways, and responses to chemotherapy. *, *p* < 0.05; **, *p* < 0.01;***, *p* < 0.001.

Given that the unique immunological status of the brain contributes to the particular tumor microenvironment of glioma, differences in the infiltration of immune cells among tumor samples and the relevance of the infiltration to prognosis and therapies deserve our exploration and study. In this article, we retrospectively analyzed the gene expression profiles and the corresponding genomic data and the patients’ follow-up information (histology, gender, age, WHO grade and overall survival, etc.,) of diffuse glioma (WHO grade II/III/IV) patients from TCGA and CGGA databases. First, we captured patient data from TCGA database and divided them into two immune Sub groups according to the difference in infiltration of immune cells. After differential expression (|log_2_ FC| > 1 and adjusted *p* value < 0.05) and co-expression analysis (|Pearson R| > 0.6 and adjusted *p* value < 0.05), 216 RBPs were defined as immune-associated RBPs (Figure 1A). Through univariate, LASSO, and multivariate Cox regression analyses, we found that the eight immune-associated RBPs prognostic signature significantly correlated with the overall survival of glioma patients in TCGA and CGGA database, respectively (Figure 1B,C). The patients were divided into high- and low-risk groups on the basis of risk score. In addition, analyses of pathway enrichment, somatic mutation, copy number variations, and immuno-/chemotherapeutic response prediction were analyzed in different risk groups (Figure 1D). Article framework and workflow have been shown in Figure 1.

**Figure 2:**
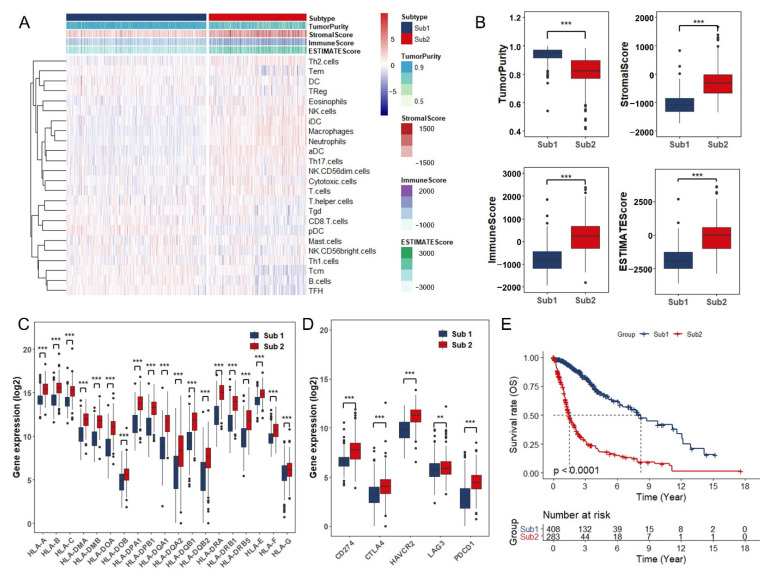
Glioma immune subtypes were identified based on the tumor-infiltrating immune cells: (**A**) Heatmap of ssGSEA scores for two subtype groups (Sub1= 408, Sub2 = 283). (**B**) Comparison of tumor purity, estimate score, immune score and stromal score between Sub1 and Sub2 in TCGA database. (**C**) The expression level of HLA family genes in Sub1 (*n* = 408) and Sub2 (*n* = 283) in TCGA database. (**D**) The expression level of CD274 (PD-L1), CTLA4, HAVCR2, LAG3 and PDCD1 (PD1) in Sub1 (*n* = 408) and Sub2 (*n* = 283) in TCGA database. (**E**) Kaplan–Meier curves of overall survival for Sub1 (*n* = 408) and Sub2 (*n* = 283) in TCGA database. **, *p* < 0.01; ***, *p* < 0.001.

Considering the richness of multiple immune cell types in glioma, ssGSEA was utilized to evaluate the infiltration of twenty-four immune-related cells based on gene expression data from TCGA (Figure 2A). The “ConsensusClusterPlus” package was applied then to divide all tumor samples into k (k = 2–9) different subtypes according to differences of infiltration. On the basis of the consensus scores, the CDF curve achieves the best partition efficiency when k = 2 (Figure S1A–C). Both of the Sub groups are associated with tumor-infiltrating immune cells. There are 408 cases in Sub1 group and 283 cases in Sub2 group. The difference between them is dominant types of immune cells. Macrophages and neutrophils are dominant in tumor-infiltrating immune cells in the Sub2 group with relatively high percentage of immune cell infiltration, while central memory T (Tcm) cells, effective memory T (Tem) cells, B cells, and follicular helper T cells (TFH) mostly infiltrated into Sub1 group (Figure 2A and Figure S1D). ESTIMATE algorithm was used to calculate Tumor Purity, ESTIMATE Score, Immune Score, and Stromal Score of the two Sub groups. With relatively low tumor purity, Sub2 group gets higher ESTIMATE, immune and stromal score when compared with Sub1 group, and the opposite results are observed in Sub1 group (Figure 2A,B). The date shown above is consistent with the results observed in the expression of human leukocyte antigen (HLA) family, which is required to present endogenous cellular antigens to circulating T cells and regulate immune response to tumors (Figure 2C). Moreover, we detected the expression of several immune checkpoint biomarkers such as programmed cell death 1 ligand 1 (PD-L1, CD274), CTLA4, Hepatitis A virus cellular receptor 2 (HAVCR2), Lymphocyte-activation gene 3 (LAG3), and PDCD1 (PD1). The expression level of these marker genes in Sub2 group was significantly higher than that in Sub1 group, indicating that more severe immune exhaustion happened in the tumors of Sub2 group (Figure 2D). In addition, we used the CIBERSORT method to verify the above results and found that macrophages, especially the immunosuppressive subtype M2 macrophages, and neutrophils cells infiltrated into Sub2 group, while more naive B cells and TFH cells remain in Sub1 group (Figure S1E). Consistently, the Kaplan–Meier curve demonstrated that patients in Sub2 group had more limited overall survival than Sub1 group (Figure 2E). These findings confirmed that the tumor-infiltrating immune cells were predominantly risk factors to the overall survival of glioma patients.

### 3.2. Identification and Functional Enrichment Analysis of Immune-Associated RBPs in Glioma Patients

**Figure 3:**
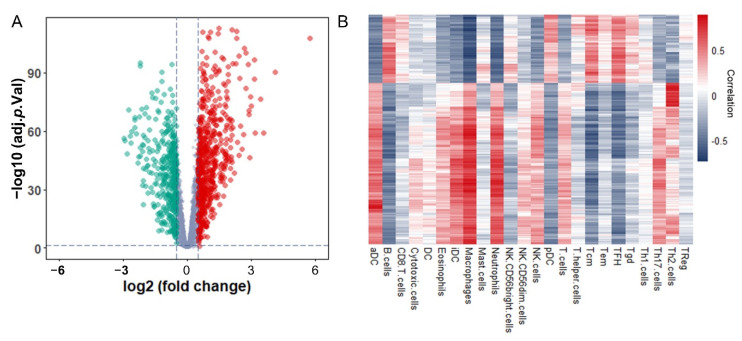
Identification and functional enrichment analysis of immune-associated RBPs in glioma patients: (**A**) Volcano plot representing the differentially expressed immune-associated RBPs between Sub1 (*n* = 408) and Sub2 (*n* = 283) in glioma. (**B**) Correlation matrix of 216 immune-associated RBPs and 24 types of tumor-infiltrating immune cells.

To sort out the immune-associated RBPs to evaluate the prognosis of glioma patients more accurately, the expression matrixes of 4127 RBPs based on website and literature reported [16] were collected from the TCGA database for further analysis. First, RBPs with differential expression between Sub1 and Sub2 groups were screened out. According to the criteria of |log_2_ FC| > 1 and adjusted *p* value < 0.05, 357 RBPs were differentially expressed between Sub1 and Sub2 groups. Among them, the expression of 228 RBPs were up-regulated and 129 were down-regulated in Sub2 group, respectively (Figure 3A and Figure S2A). Next, the correlation analysis between the expression of those 357 RBPs and ssGSEA score of 24 tumor-infiltrating immune cells was performed in all glioma patients. The RBP that its expression value was correlated with at least one type of immune cells (|Pearson R| > 0.6 and adjusted *p* value < 0.05) was defined as an immune-associated RBP. A total of 216 differentially expressed RBPs were defined as immune-associated RBPs for subsequent studies (Figure 3B and Figure S1).

Next, GO and KEGG pathway enrichment analyses were performed to explore the potential functions of these immune-associated RBPs. GO analysis indicated that these RBPs were categorized into several essential biological processes, including interferon-gamma-mediated signaling pathway, extracellular matrix organization, type I interferon signaling pathway, leukocyte migration, and negative regulation of viral genome replication, which further verified the significant correlation between RBPs and immunoreaction. In terms of cellular component, the majority of genes were located in extracellular exosome, focal adhesion, cell surface, cytoplasm, and cytosol. In molecular function enrichment analysis, these RBPs were enriched in double-stranded RNA binding, poly(A) RNA binding, protein binding, RNA binding and glycoprotein binding (Figure S2B). Additionally, KEGG pathway analysis demonstrated that the most significant pathways were ECM-receptor interaction, focal adhesion, regulation of actin cytoskeleton, leukocyte transendothelial migration, and proteoglycans in cancer (Figure S2C).

### 3.3. Identification and Assessment of an Immune-Associated RBPs Prognostic Signature for Overall Survival in Glioma.

**Figure 4:**
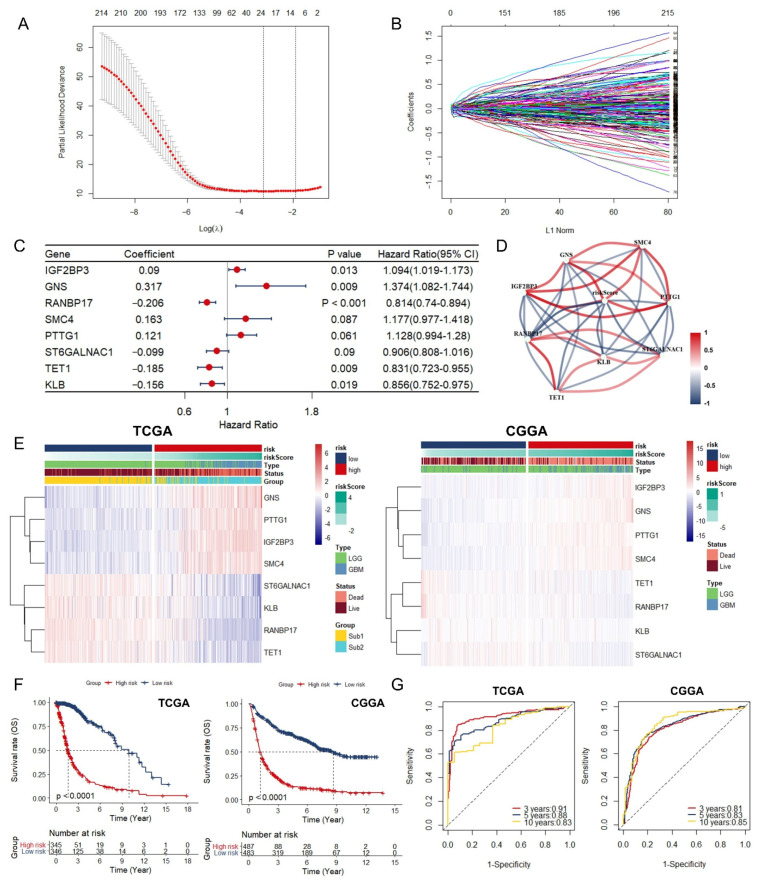
Identification and assessment of an immune-associated RBPs prognostic signature for overall survival in glioma: (**A**) Robust prognostic genes identified through LASSO regression algorithm. (**B**) Distribution of LASSO coefficients for 216 genes in the 10-fold cross validation. (**C**) Forest plot of the prognostic ability of the eight immune-related RBPs included in the prognostic signature. (**D**) A correlation network involving the eight immune-associated RBPs and risk score in TCGA. (**E**) Heatmap and clinicopathologic features of high- and low-risk groups based on the eight immune-associated RBPs in the TCGA (left, high risk = 345, low risk = 346) and CGGA (right, high risk = 487, low risk = 483) database. (**F**) Kaplan–Meier curves showing different overall survival of patients in low- and high-risk groups. (**G**) ROC curves of immune-associated RBPs for predicting the 3/5/10-year overall survival in TCGA (**left**) and CGGA (**right**) database.

Univariate Cox regression method was applied first to investigate the prognostic significance of these 216 RBPs, and all of them were verified as prognosis-related RBPs. In order to avoid the overfitting of prognostic signature, we performed LASSO regression analysis on these RBPs next by 10-round cross-validation, and found 19 candidates that were closely associated with the prognosis of glioma patients, including: MSN, STEAP3, IGF2BP3, NSUN6, CTSC, GNS, HMGN5, RANBP17, SMC4, DUSP9, GLUD1, PTTG1, MCAM, KHDRBS2, GGH, ST6GALNAC1, TET1, KLB, ZNF483 (Figure 4A,B). Furthermore, stepwise multiple Cox regression analysis narrowed down the 19 candidates to eight immune-associated RBPs which were used to establish the predictive model. The risk score of each patient was calculated based on the coefficients (Exp: Expression) (Figure 4C):

Risk score = 0.09*ExpIGF2BP3 + 0.317*ExpGNS – 0.206*ExpRANBP17 + 0.163*ExpSMC4 + 0.121*ExpPTTG1 – 0.099*ExpST6GALNAC1 – 0.185*ExpTET1 – 0.156*ExpKLB

In TCGA database, the correlation network showed that IGF2BP3, GNS, SMC4, and PTTG1 were positively correlated with the risk score, while RANBP17, ST6GALNAC1, TET1, and KLB were negatively correlated with the risk score (Figure 4D). Also, relatively higher risk scores were observed in the Sub2 group (Figure S3A).

Based on the median of risk score, the glioma patients whose risk scores were higher than the median were defined as high-risk group and low-risk group was defined in a similar way in TCGA and CGGA database, respectively (Figure 4E). High expression of IGF2BP3, GNS, SMC4, and PTTG1 was noticed in high-risk group, while high expression of RANBP17, ST6GALNAC1, TET1, and KLB in low-risk group (Figure 4E). Meanwhile, Kaplan–Meier curve indicated that high-risk group conferred worse prognosis (Figure 4F), which was verified in low grade glioma (LGG) group, glioblastoma multiforme (GBM) group, TCGA-Sub1, and TCGA-Sub2 (Figure S3B,C). Finally, the time-dependent ROC presented relatively excellent performance in survival prediction. The AUCs for each ROC was 0.91 (TCGA, 3 year), 0.88 (TCGA, 5 year), 0.83 (TCGA, 10 year), 0.81 (CGGA, 3 year), 0.83 (CGGA, 5 year), and 0.85 (CGGA, 10 year) (Figure 4G).

### 3.4. Construction of Integrated Model to Predict Survival of Glioma Patients

Next, univariate and multivariate Cox regression analyses were performed to evaluate the prognostic significance of the eight immune-associated RBPs prognostic signature combined with various clinicopathologic parameters in the TCGA database. Univariate analysis indicated that age, sex, grade, ATRx, IDH, MGMT promoter, TERT, immune subtypes, and the prognostic signature were significantly associated with overall survival. Subsequent multivariate analysis uncovered that age, IDH status, and the eight immune-associated RBPs prognostic signature were notably correlated with overall survival. Therefore, our prognostic signature based on eight immune-associated RBPs was proven to be an independent prognostic indicator for glioma in TCGA database (Table 1), which have been verified in CGGA database (Table S2).

**Figure 5:**
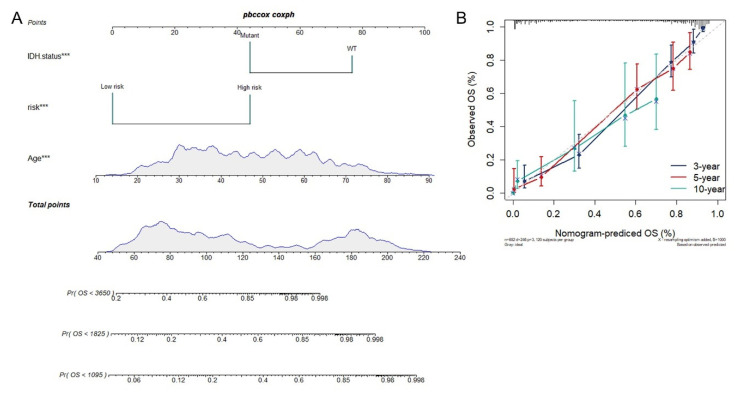
Construction of integrated models to predict survival in glioma: (**A**) Nomogram for predicting the 3/5/10-year overall survival in glioma patients. (**B**) Calibration curve for the prediction of 3/5/10-year overall survival. ***, *p* < 0.001.

To construct a glioma prognosis model suitable for clinical use, we established a prognostic nomogram to predict 3-, 5-, 10-year overall survival based on the stepwise Cox regression model in TCGA database. Age, IDH status and Risk score had been included in the prediction model (Figure 5A). The C-index of the nomogram was 0.849 (95% CI, 0.827 to 0.871). Nomogram prediction and actual observation in TCGA database reached an excellent agreement at the 3-, 5-, and 10-year survival probability after calibration (Figure 5B).

### 3.5. Estimation of Tumor-Infiltrating Immune Cells and Prediction of Therapeutic Response to Immune Checkpoint Inhibitors

**Figure 6:**
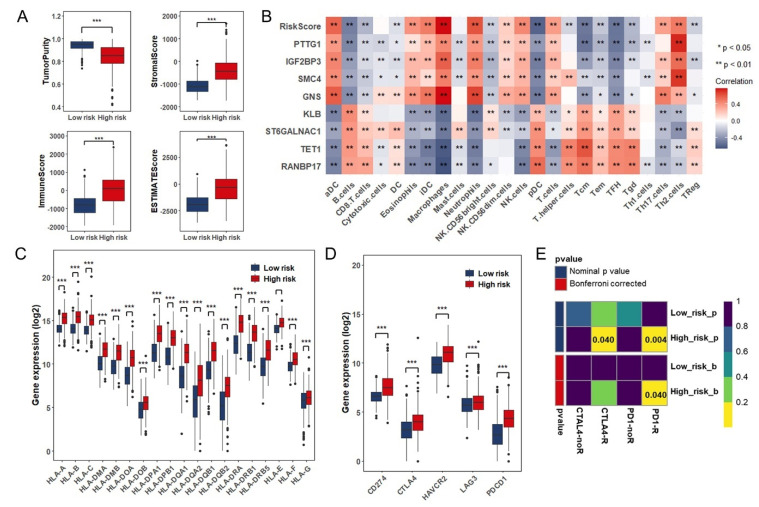
Estimation of tumor-infiltrating immune cells and prediction of responses to immune checkpoint inhibitors: (**A**) Comparison of tumor purity, estimate score, immune score, and stromal score between low-risk group (*n* = 346) and high-risk group (*n* = 345) in TCGA database. (**B**) Correlation matrix of risk score, eight immune-associated RBPs and 24 types of tumor-infiltrating immune cells. (**C**) The expression level of HLA family genes in low- and high-risk groups in TCGA database. (**D**) The expression level of CD274 (PD-L1), CTLA4, HAVCR2, LAG3, and PDCD1 (PD1) in low- and high-risk groups in TCGA database. (**E**) Sensibility of response to PD1 and CTLA4 inhibitors of patients in low- and high-risk groups. *, *p* < 0.05; **, *p* < 0.01; ***, *p* < 0.001.

Considering that the prognostic model was developed based on immune-associated RBPs, we consequently investigated whether this model was linked to the tumor immune microenvironment. Through ESTIMATE algorithm, the high-risk group had significantly lower tumor purity and higher ESTIMATE Score, immune score and stromal score compared to low-risk group (Figure 6A). A detailed Spearman correlation analysis is showed in Figure 6B. Among these immune cells, a high-risk score was positively associated with dendritic cells, macrophages, and neutrophils, whereas negatively associated with B cells and T cells (Figure 6B,C). Additionally, we detected the expression of HLA family and found they were significantly increased in high-risk group (Figure 6D). Also, we also investigated the relationship between the risk index and immune checkpoint and then discovered that risk score was positively correlated with the expression of CD274 (PD-L1), CTLA4, HAVCR2, LAG3, and PDCD1 (PD1) (Figure 6D). David A Reardon [31] discovered that combination therapy of anti-CTLA-4 plus anti-PD-1 resulted in changes of immune landscape that the proportion of activated CD8+ cells as well as natural killer cells increased and suppressive immune cells decreased in the tumor microenvironment, which achieved the therapeutic effect and prolonged tumor-free survival in murine glioblastoma model. Based on the background hereinbefore, we wondered whether there are differences in response to immunotherapy between low- and high-risk groups. Next, subclass mapping algorithm was used to predict the possibility of effective responses to immunotherapy. Comparing the expression profile of low- and high-risk groups with another published dataset containing 47 patients with melanoma that responded to immunotherapy [32], we found that high-risk group tended to respond effectively to immunotherapy such as anti–PD-1 (Bonferroni corrected *p* = 0.040) and anti-CTLA-4 therapy (*p* = 0.040) (Figure 6E).

### 3.6. Somatic Mutation and Copy Number Variations in Different Risk Groups

**Figure 7:**
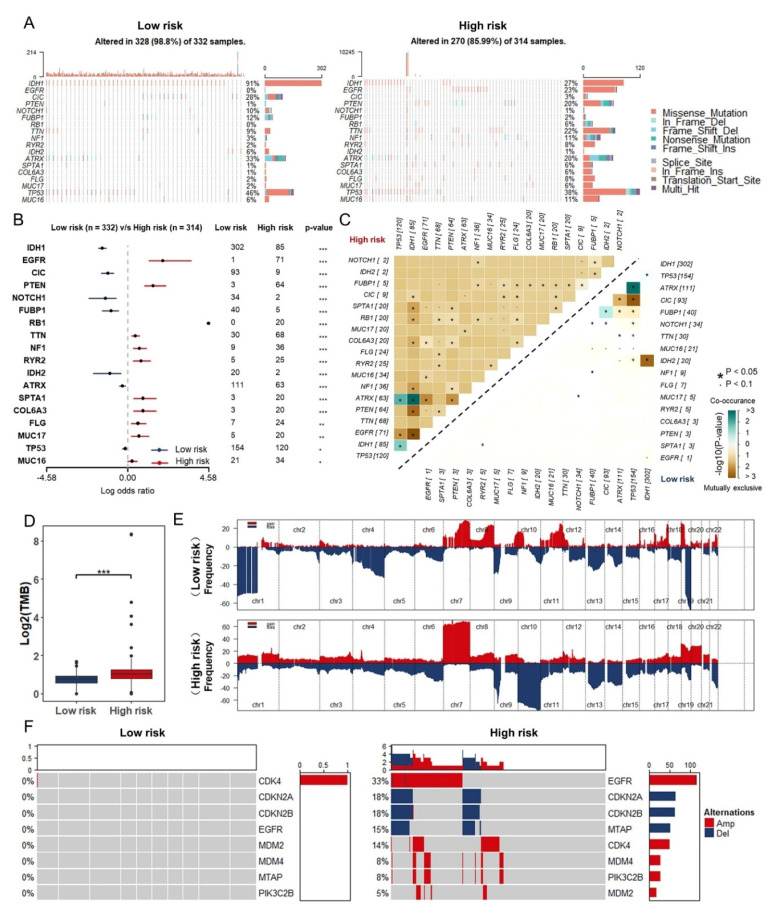
Somatic mutation and CMVs in different risk groups: (**A**) Heatmap of somatic mutations in different risk groups. (**B**) The differently mutated genes between low- and high-risk groups. (**C**) Heatmap of co-occurrence and mutually exclusive mutations of the differently mutated genes in low- and high-risk group. (**D**) TMB in low- and high-risk groups in TCGA database. (**E**) Detection and comparison of significant amplifications and deletions of copy number between low- and high-risk group. (**F**) Comparison of CNVs between low- and high-risk groups in TCGA database. *, *p* < 0.05; **, *p* < 0.01; ***, *p* < 0.001.

Previous studies have shown that somatic mutations in tumor-associated genes (EGFR, IDH1, TP53, NF1, and etc.,) and large regions of CNVs were regarded as major roles in tumorigenesis and the development of glioma [33,34,35]. Therefore, we analyzed the somatic mutations and CNVs from TCGA database to explore the genomic alterations in different risk groups. With the threshold of *p* value < 0.05 (Fisher’s exact test) and the mutation frequency exceeding 20 in one cohort, somatic mutations in different genes were detected in different risk groups. Mutational genes with high frequency were IDH1, CIC, NOTCH1, FUBP1, and IDH2 in the low-risk group, while EGFR, PTEN, RB1, TTN, and NF1 in the high-risk group (Figure 7A,B). Furthermore, we investigated the distribution of co-occurrence and mutually exclusive mutations in groups, and found that a unique ATRX-IDH1 co-occurrence and the majority of exclusive mutations had been discovered in high-risk group (Figure 7C) with significantly elevated tumor mutational burden (TMB) (Figure 7D).

As for copy number variations, we performed the GISTIC2.0. High frequency deletion of regions on chromosome (Ch) 1, 4, and 19 has been discovered in low-risk group, which is consistent with the existing analysis of clinical samples that the glioma patients with 1p/19q co-deletion tend to have better prognosis [36]. In contrast, widely amplified regions on Ch7 and frequent deletion of regions on Ch10 have been noticed in high-risk group (Figure 7E). Taken together, more emphasis was given to the genes whose copy number variation are predominant. EGFR, CDK4, MDM4, PIK3C2B, and MDM2 were widely amplified, while CDKN2A and CDKN2B were deleted in the high-risk group (Figure 7F).

### 3.7. GSVA and GSEA for Different Risk Groups

**Figure 8:**
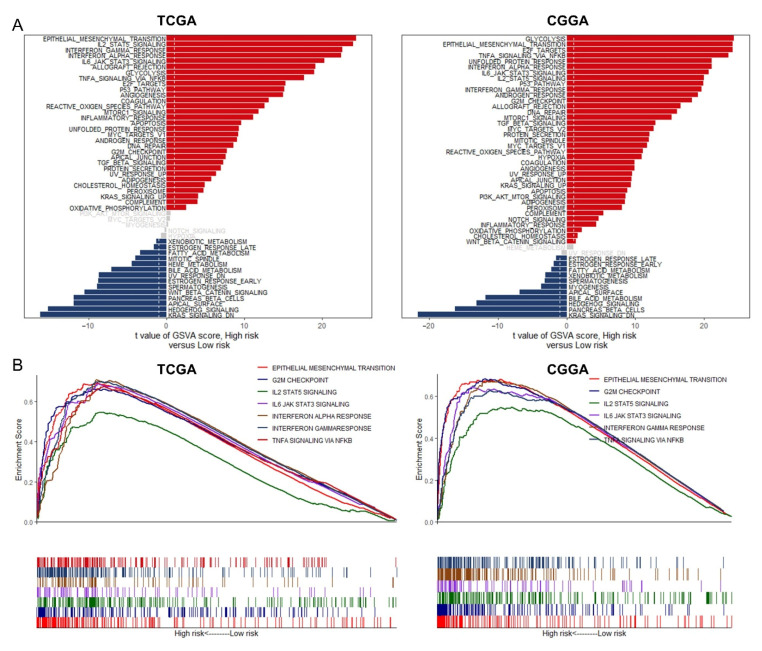
The GSVA and the GSEA for different risk groups: (**A**) The enrichment of GSVA between the low- and high-risk group and the significantly differentially expression pathways validated by “limma” analysis. (**B**) GSEA plot of major gene sets coactivated in both TCGA and CGGA.

For further investigation into the potential biological pathways and processes in the two groups with different degrees of risk, we conducted GSVA and GSEA in TCGA and CGGA database. As for the results of GSVA enrichment (Figure 8A), we found that critical pathways associated with tumorigenesis including epithelial mesenchymal transition (EMT), angiogenesis, apoptosis, DNA repair, G2/M checkpoint, and apical junction have been enriched in high-risk group. The immune-related pathways such as IL2-STAT5 signaling, interferon gamma response, interferon alpha response, and IL6-JAK-STAT3 signaling were also enriched in this group. Moreover, the GSEA revealed parallel results that tumor-associated pathways and immune-related pathway were extremely enriched in the high-risk group (Figure 8B). The detail results of GSVA and GSEA can be found in Tables S3 and S4.

### 3.8. The Role of the Eight Immune-Associated RBPs Signature in Predicting the Sensitivity to Chemotherapeutic Agents

**Figure 9:**
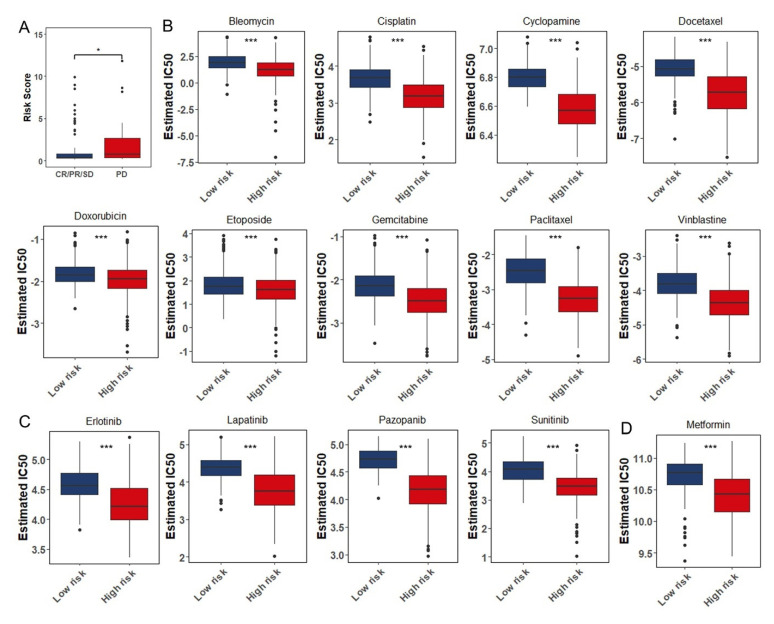
The role of the eight immune-related RBPs signature in predicting the sensitivity to chemotherapeutic agents: (**A**) The risk score of each group was calculated and verified to be statistically significant between effective group (*n* = 100, CR: complete response; PR: partial response; SD: stable disease) and ineffective group (*n* = 31, PD: progressive disease). (**B**) The box plots of the estimated IC_50_ for Bleomycin, Cisplatin, Cyclopamine, Docetaxel, Doxorubicin, Etoposide, Gemcitabine, Paclitaxel, and Vinblastine. (**C**) The box plots of the estimated IC_50_ for Erlotinib, Lapatinib, Pazopanib, and Sunitinib. (**D**) The box plot of the estimated IC_50_ for Metformin. Low risk (*n* = 346); High risk (*n* = 345); *, *p* < 0.05; ***, *p* < 0.001.

Neurosurgical resection followed by radiotherapy with subsequent chemotherapy is the standard treatment of glioma so far [37]. Temozolomide has been considered to be the first-line chemotherapeutic agent in the treatment of glioma. According to the date from TCGA, glioma patients treated with temozolomide, as a previous study has selected [38], were divided into effective group (CR: complete response; PR: partial response; SD: stable disease) and ineffective group (PD: progressive disease). The risk score of each group was calculated and verified to be statistically significant between two groups (Figure 9A). The result was that relatively high-risk scores of the temozolomide ineffective group coincided with the aforementioned research. However, there were still some patients who might be insensitive to temozolomide or failed temozolomide therapy due to resistance. So, we used “pRRophetic” package to estimate the chemotherapeutic sensitivity of 138 drugs in different risk groups [28]. We selected nine chemotherapeutic agents (Bleomycin, Cisplatin, Cyclopamine, Docetaxel, Doxorubicin, Etoposide, Gemcitabine, Paclitaxel, and Vinblastine) that might benefit patients in high-risk group and estimated their IC_50_ values if they were used to treat patients (Figure 9B). As is known in result 6 (Figure 7) that high frequency of mutation and amplified copy number variation of EGFR existed and the angiogenesis pathway was enriched in high-risk group. Small-molecule inhibitors such as Erlotinib and Lapatinib targeting EGFR as well as Pazopanib and Sunitinib targeting VEGFR were further screened out, and the estimated IC_50_ values of these drugs were significantly reduced in the high-risk group (Figure 9C). Besides, the patients from high-risk group were predicted to be more sensitive to Metformin (Figure 9D).

## 4. Discussion

Glioma derived from neuroepithelium is regarded as the most common and lethal type of primary brain tumor with an extremely poor prognosis, accounting for about 30 percent of all CNS tumors and 80 percent of all primary malignant brain tumors [1]. GBM accounts for half of glioma cases. Its highly aggressive nature limits the 5-year survival rate to only 4.7% [39] and the median survival of GBM patients to less than 15 months [40]. According to the WHO classification of tumors of the CNS, malignant adult diffused gliomas are classified into grades II to IV based on histologic features. In the 2016 edition, IDH mutation and chromosomal co-deletion 1p/19q are integrated into the traditional glioma [2]. Even though complicated classification criteria are applied for prognostic evaluation, the prognostic survival boundaries between subtypes are still fuzzy. At present, an accurate and operable prognostic model is urgently needed in clinic. This paper timely provides a prognostic signature with high accuracy and great value to clinical drug application based on TCGA and various algorithms.

Most recently, great breakthroughs have been made by immune checkpoint inhibitors which dramatically change the treatment landscape for patients with cancer [41]. Yet the clinical application of checkpoint inhibitors in glioma is up against tremendous challenges because of the “cold phenotype” of glioma. For finding more effective immunotherapy, a deeper understanding of the immune characteristics of glioma is particularly important. A variety of immune cells including macrophages, neutrophils, CD4+ helper T (Th) cells, regulatory T (T reg) cells, CD8+ cytotoxic T lymphocytes (CTLs), and natural killer (NK) cells are present in glioma microenvironment [4]. In this study, we retrospectively analyzed the transcriptome data of glioma patients who were further classified into Sub1 and Sub2 according to differences in immune cell infiltration. Macrophages, especially M2 macrophages, and neutrophils are dominant in tumor-infiltrating immune cells in the Sub2 with relatively high percentage of immune cell infiltration, while Tcm, Tem, B and TFH cells mostly infiltrated into Sub1 group. Besides, with relatively low tumor purity, Sub2 gets higher ESTIMATE, immune and stromal score when compared with Sub1, and the opposite results were observed in Sub1 group.

Tumor-derived cytokines and chemokines are able to reprogram those immune cells into tumor-associated phenotypes which therefore have profound effects on the progression and therapeutic resistance. These processes, mediated by multiple RBPs, coordinately control inflammatory gene expression, providing an efficient way to rapidly respond to inflammatory mediators and facilitate a coordinated systemic immune and control the overall status of cells. Given the critical roles of RBPs in immune regulation, potentially pervasive connections between immune response and RNA regulation are just waiting for us to discover. Differentially expressed RBP between Sub1 and Sub2 groups were selected out for further screening, analysis, and construction of a prognostic signature composed of eight immune-associated RBPs including canonical and non-canonical ones. Canonical RBPs work by binding to conserved sequence motifs in their target mRNAs via combinations of structurally well-defined RNA-binding domains (RBDs) [42]. Classic RBDs include the RNA recognition motif, the K-homology, DEAD/DEAH helicase and zinc-finger domains [8]. Besides, non-canonical RBPs refers to those proteins which have not been proved to have classic RBDs or the established domains by direct experimental evidence but have RNA-binding activity. In our prognostic signature, the IGF2BP3 (insulin like growth factor 2 mRNA-binding protein 3) and RANBP17(RAN-binding protein-17) are classic RBPs. IGF2BP3 primarily plays an oncogenic role in various cancers. Over the past few years, studies have increasingly documented the contribution of IGF2BP3 to tumor cell proliferation, blocking apoptosis, favoring stemness, promoting migration and drug resistance, and IGF2BP3 overexpression has been widely associated with adverse patient outcomes in many different tumors [43]. RANBP17 plays a key role in nuclear localization signal-dependent protein import. The limited research has shown that high expression of RANBP17 implied a relatively good prognosis [44].

Next, according to the risk score of each patient figured out based the prognostic signature composed of eight immune-associated RBPs, we grouped glioma patients into two risk (low-risk and high-risk) groups. In TCGA and CGGA databases, we found that the patients with high risk scores tended to have poor overall survival. In order to explore the feasibility of the prognostic signature in clinical application, we performed the univariate and multivariate Cox analyses on the risk score and the clinical indexes of glioma cancer patients, such as age, sex, grade, and etc. Here, we confirmed that the eight immune-associated RBPs prognostic signature could be an independent prognostic factor in patients with glioma. In addition, nomogram including our prognostic signature showed the best performance in predicting 3-, 5- and 10-year OS, which might help guide the individualized treatment of glioma patients.

Furthermore, we researched on the relation of the eight immune-associated RBPs prognostic signature to immune microenvironment. The high-risk group had significantly lower tumor purity and higher ESTIMATE Score, immune score and stromal score compared to low-risk group. For the type of infiltrating immune cells, high-risk scores were mostly positively associated with the degree of infiltration of dendritic cells, macrophages, and neutrophils, whereas negatively associated with B cells and T cells. In the immune microenvironment of glioma, tumor-associated macrophages (TAMs) are major tumor-infiltrating immune cells including marrow-derived macrophages and brain resident microglia [45]. Although macrophages should be able to kill tumor cells, immunosuppressive microenvironment most often polarizes TAMs into tumor-supporting cells (M2-like macrophages) rather than pro-inflammatory subtypes (M1-like macrophages), which promote immunosuppression, angiogenesis, and extracellular matrix [46,47]. Contrary to the pro-inflammatory function during infections, tumor-associated neutrophils (TANs) promotes tumor progression malignancy by mediating angiogenesis [48]. When it comes to a variety of T cells, as the key component of the anti-tumor immune response, tumor-infiltrating lymphocytes represented by CD4+ Th cells and CD8+ CTLs are only present in remarkably low numbers in the CNS. Moreover, T reg cells in the glioma microenvironment mediate immunosuppressive effects by exhausting CTLs [49]. CD8+ Tcm cells derived from naive T cells stimulated by antigens are responsible for long-term memory of immune response. When received the same stimulus, a large number of CD8+ Tem cells against the same antigens can be cloned from CD8+ Tcm cells and then differentiate into CD8+ effector T cells that are powerful tumor killers. Klebanoff CA et al. [50] first demonstrated that tumor-reactive CD8+ Tcm cells have superior anti-tumor ability. Besides, NK cells are usually in a nonfunctional state due to excessive immunosuppression in glioma [4]. Additionally, we detected the expression of HLA family and found they were significantly increased in high-risk group. Also, we also investigated the relationship between the risk score and immune checkpoint biomarkers and then discovered that the expression of CD274 (PD-L1), CTLA4, HAVCR2, LAG3, and PDCD1 (PD1) was increased in the high-risk group. The prediction of the anti-PD-1 and anti-CTLA-4 treatment response showed that the patients in high-risk group tended to respond effectively to immunotherapy.

Based on the risk score, we further explored TMB, somatic mutation, CNVs, and enrichment pathway. EGFR was one of the first oncogenes identified in GBM and remains one of the most attractive therapeutic targets. Genomic alterations in EGFR are present in 57% of patients and are strikingly diverse, including gene amplification, rearrangements, and point mutations [51]. In the high-risk group, we found EGFR with high frequency mutation and wide amplification. In addition, GSVA analyses showed that angiogenesis pathway was enriched in the high-risk group. In the tumor microenvironment, IL-6/JAK/STAT3 signaling induced the expression of factors that promotes angiogenesis such as VEGF and invasiveness such as matrix metalloproteinases [52], while strongly suppressing the antitumor immune response [53]. TAMs have been described as potent EMT inducers in numerous independent studies. TAMs accordingly produce multiple growth factors (HGF, EGF, TGF, PDGF, etc.,) and inflammatory cytokines (IL-1β, IL-6, and TNF-α) that each can induce EMT in cancer cells [54,55]. In predicting the sensitivity to chemotherapeutic agents, small-molecule inhibitors such as Erlotinib and Lapatinib targeting EGFR as well as Pazopanib and Sunitinib targeting VEGFR were further screened out, and the estimated IC_50_ values of these drugs were significantly reduced in the high-risk group. Additionally, the combination of a PD-1 inhibitor and VEGF inhibitor was found to be tolerable and promising in animal and clinical models [56]. This study demonstrated that a novel signature constructed by immune-associated RBPs was valuable in predicting the survival of patients with glioma and might help in directing the selection of chemotherapeutic agents and distinguishing those who could benefit from anti-tumor immunotherapy.

Our study still has several limitations. First, we only used the CGGA databases to validated the prognostic risk model and therefore more independent glioma database should be included to confirm the predictive capacity of our findings. Second, how do the immune-associated RBPs identified in this study, especially eight RBPs in our prognostic signature, regulate the infiltration of immune cell should be confirmed by in vitro and in vivo experiments and deserve further study.

## 5. Conclusions

In summary, our study provides broad molecular signatures for further functional and therapeutic investigations of RBPs in glioma microenvironment, and represents a potential systemic approach to characterize key proteins in glioma pathogenesis and therapeutic responses.

## Figures and Tables

**Table 1:** Univariate and multivariate Cox regression analysis of clinical pathologic features in TCGA database.

	Univariate Analysis		Multivariate Analysis	
	HR (95% CI)	*p* Value	HR (95% CI)	*p* Value
Age ≥ Median vs. < Median)	2.891 (2.38–3.511)	<0.001	1.818 (1.186–2.788)	0.006
Sex (Male vs. Female)	1.189 (1.001–1.412)	0.049	1.262 (0.917–1.737)	0.154
Grade (WHO IV vs. WHO II~III)	4.729 (3.913–5.716)	<0.001	1.402 (0.904–2.173)	0.131
ATRX.status (WT vs. Mutant)	1.911 (1.528–2.389)	<0.001	1.095 (0.623–1.924)	0.754
IDH.status (WT vs. Mutant)	5.032 (4.12–6.146)	<0.001	2.28 (1.256–4.139)	0.007
MGMT.promoter (Methylated vs. Unmethylated)	2.333 (1.924–2.829)	<0.001	0.854 (0.573–1.272)	0.436
TERT.promoter (WT vs. Mutant)	0.588 (0.44–0.785)	<0.001	0.948 (0.558–1.612)	0.845
Immune subtype (Sub2 vs. Sub1)	3.262 (2.708–3.929)	<0.001	1.414 (0.912–2.195)	0.122
Risk (High vs. Low)	4.335 (3.468–5.421)	<0.001	1.897 (1.147–3.138)	0.013

## Data Availability

The data presented in this study are available in the article and Supplementary Materials.

## Supplementary Materials

The following are available online at https://www.mdpi.com/article/10.3390/cancers13071730/s1. Figure S1: Glioma subtypes identified based on tumor-infiltrating immune cells. Figure S2: Identification and functional enrichment analysis of immune-associated RBPs in glioma patients. Figure S3: Identification and assessment of the eight immune-associated RBPs prognostic signature for overall survival in glioma. Table S1: The list of 216 immune-associated RBPs, Table S2: Univariate and multivariate Cox regression analysis of clinical pathologic features in CGGA dataset, Table S3: The GSVA significantly differentially expression pathway between the low- and high-risk group in TCGA and CGGA, Table S4: The GSEA significantly differentially expression pathway between the low- and high-risk group in TCGA and CGGA.
