# Supplementary material for: Identification and Validation of an Immune-Associated RNA-Binding Proteins Signature to Predict Clinical Outcomes and Therapeutic Responses in Glioma Patients
Source: Cancers (Basel). 2021 Apr 6;13(7):1730. doi: 10.3390/cancers13071730 (PMC8038676; doi:10.3390/cancers13071730)
Supplement: Supplementary file 1 [file cancers-13-01730-s001.zip › cancers-1152160 supplementary/Supplementary Figures and Tables/Supplementary Figures.docx]

Article

Identification and Validation of an Immune-Associated RNA-Binding Proteins Signature to Predict Clinical Outcomes and Therapeutic Responses in Glioma Patients

Ruotong Tian ^1,†^, Yimin Li ^2,†^, Qian Liu ^1^ and Minfeng Shu ^1,^*

| **Citation:** Tian, R.; Li, Y.; Liu, Q.; Shu, M. Identification and  Validation of an Immune-Associated RNA Binding Proteins Signature to Predict Clinical Outcomes and  Therapeutic Responses in Glioma Patients. *Cancers* **2021**, *13*, x. https://doi.org/10.3390/xxxxx  Academic Editor: David Ashley  Received: 4 March 2021  Accepted: 31 March 2021  Published: date  **Publisher’s Note:** MDPI stays neutral with regard to jurisdictional claims in published maps and institutional affiliations.  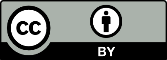  **Copyright:** © 2021 by the authors. Submitted for possible open access publication under the terms and conditions of the Creative Commons Attribution (CC BY) license (http://creativecommons.org/licenses/by/4.0/). |
| --- |

^1^ Department of Pharmacology, School of Basic Medical Sciences, Shanghai Medical College,
Fudan University, No.131 Dong’an Road, Xuhui District, Shanghai 200032, China;
20211010075@fudan.edu.cn (R.T.); 18211010067@fudan.edu.cn (Q.L.)

^2^ Department of Pathology, Fudan University Shanghai Cancer Center, No.270 Dong’an Road, Xuhui District, Shanghai 200032, China; 176501028@csu.edu.cn

* Correspondence: minfeng_shu@fudan.edu.cn; Tel.: +86-21-54237380-2

† These authors contributed equally to this work.

Supplementary
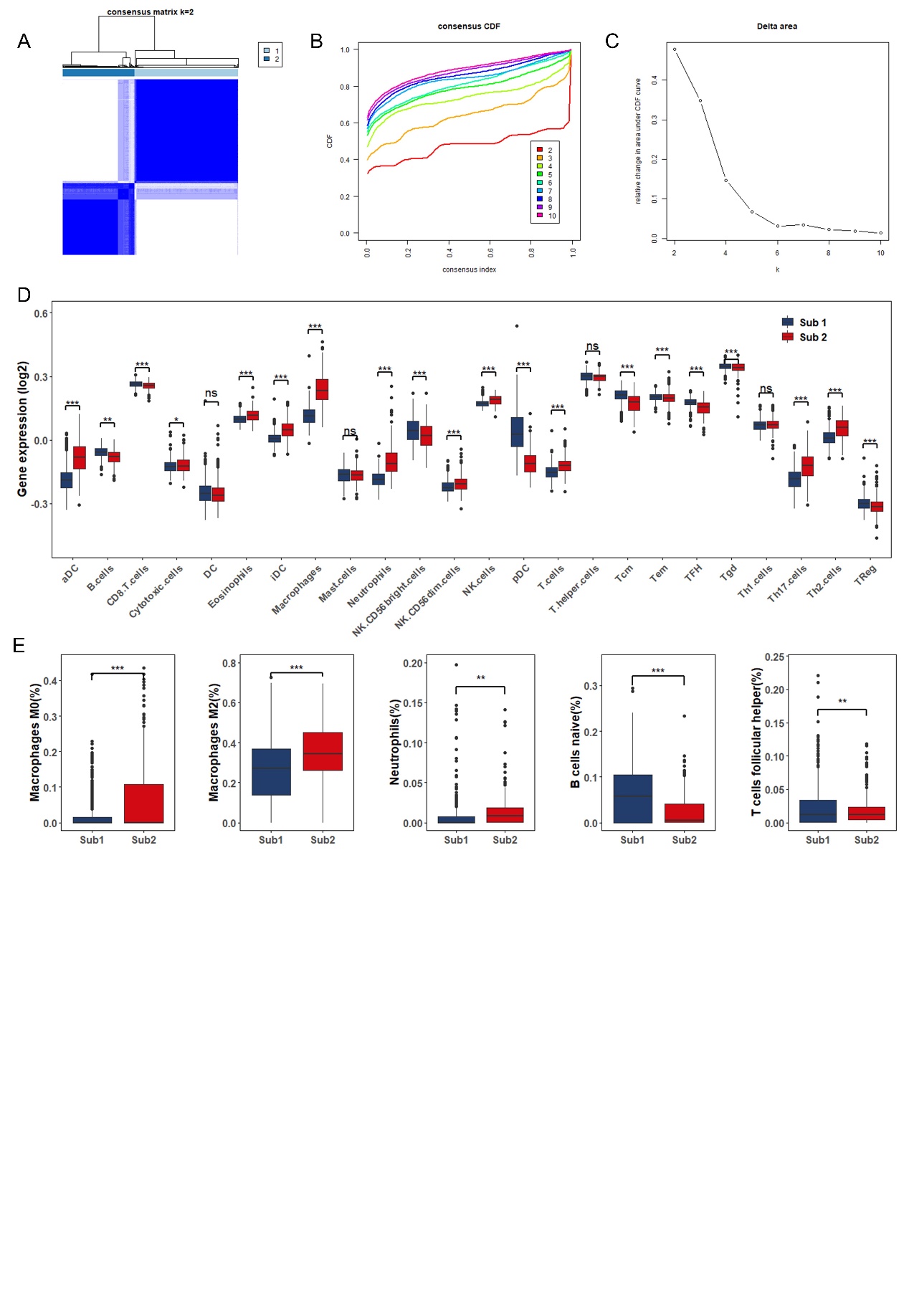


**Figure S1.** Glioma subtypes identified based on tumor-infiltrating immune cells: (A) Consensus clustering matrix for glioma samples when k = 2. (B) CDF plot showing a real random variable of its probability distribution based on consensus scores for each k (from 2 to 10, indicated by different colors). (C) Delta area curve. (D) Comparison of 24 types of tumor-infiltrating immune cells between Sub1 and Sub2 in TCGA database. (E) The infiltrating levels of the Macrophages M0, Macrophages M2, Neutrophils cells, naive B cells and follicular helper T cells in Sub1 and Sub2. **, P<0.01; ***, P<0.001.


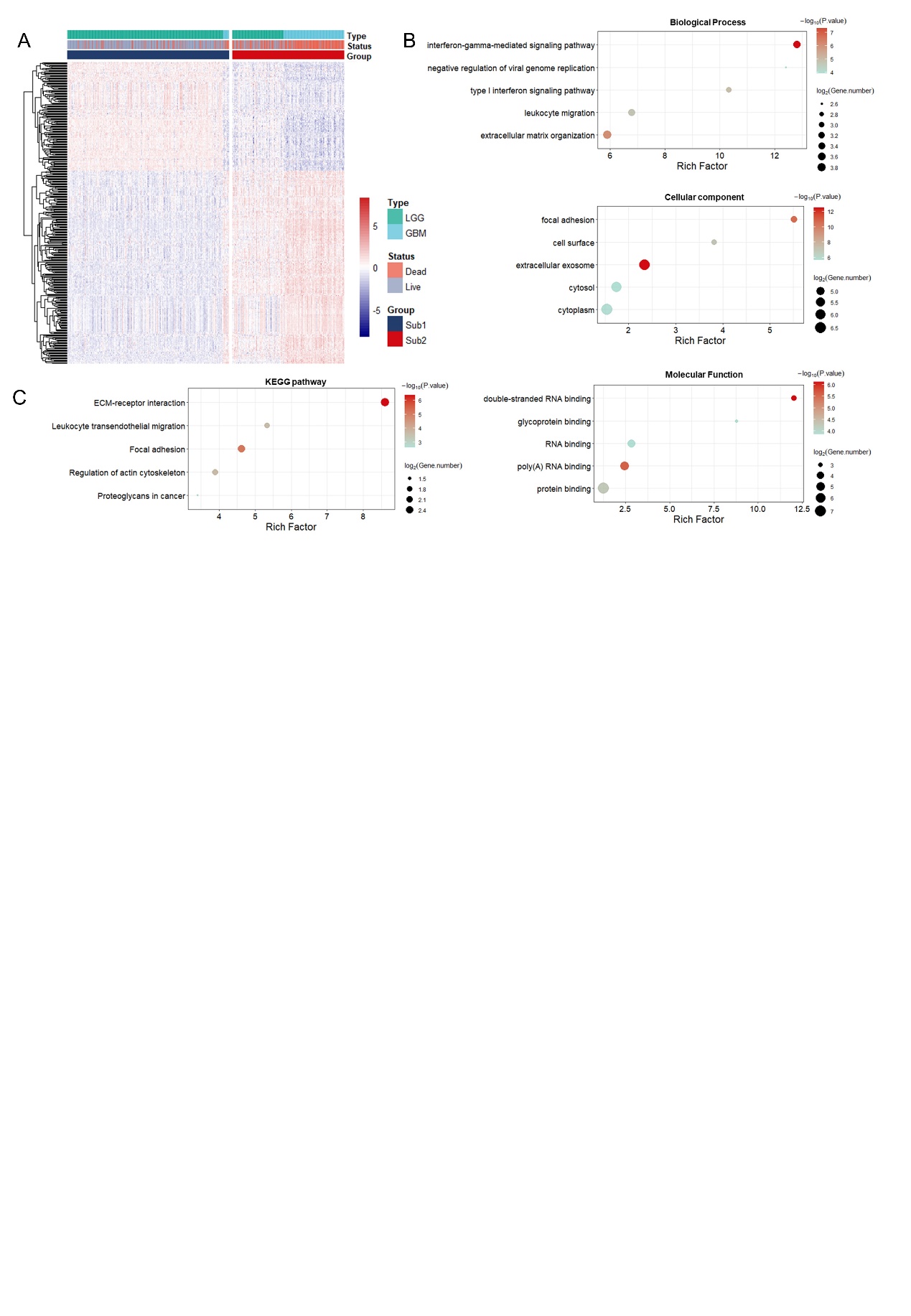


**Figure S2.** Identification and functional enrichment analysis of immune-associated RBPs in glioma patients: (A) Heatmap plot representing the differentially expressed immune-associated RBPs between Sub1 and Sub2 in glioma. (B) GO enrichment analysis of immune-associated RBPs based on Biological Processes (top), Cellular Component (middle) and Molecular Function (bottom). Top 5 enriched pathways were displayed on the figure. (C) Visualization of the top 5 enriched KEGG pathways by immune-associated RBPs.


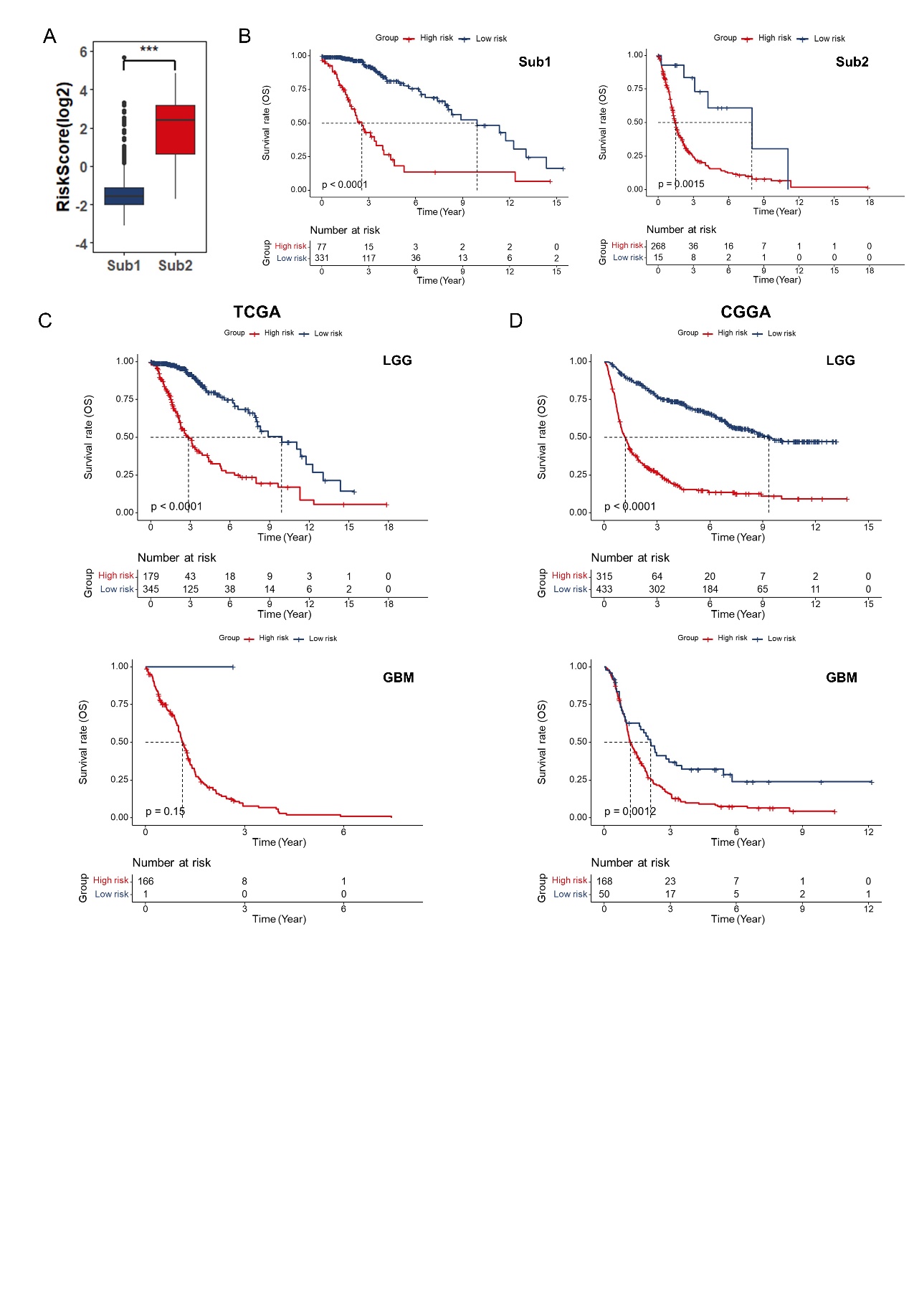


**Figure S3.** Identification and assessment of the 8 immune-associated RBPs prognostic signature for overall survival in glioma. (A) Distribution of risk scores in Sub1 and Sub2. (B) The prognosis values of immune-associated RBPs signature in Sub1 (left) and Sub2 (right) in TCGA database. (C) The prognosis values of immune-associated RBPs signature in LGG (top) and GBM (bottom) in TCGA database. (D) The prognosis values of immune-associated RBPs signature in LGG (top) and GBM (bottom) in CGGA database.
