# Supplementary material for: Identification and Validation of an Immune-Associated RNA-Binding Proteins Signature to Predict Clinical Outcomes and Therapeutic Responses in Glioma Patients
Source: Cancers (Basel). 2021 Apr 6;13(7):1730. doi: 10.3390/cancers13071730 (PMC8038676; doi:10.3390/cancers13071730)
Supplement: Supplementary file 1 [file cancers-13-01730-s001.zip › Supplementary Figures and Tables/Supplementary Figures.docx]

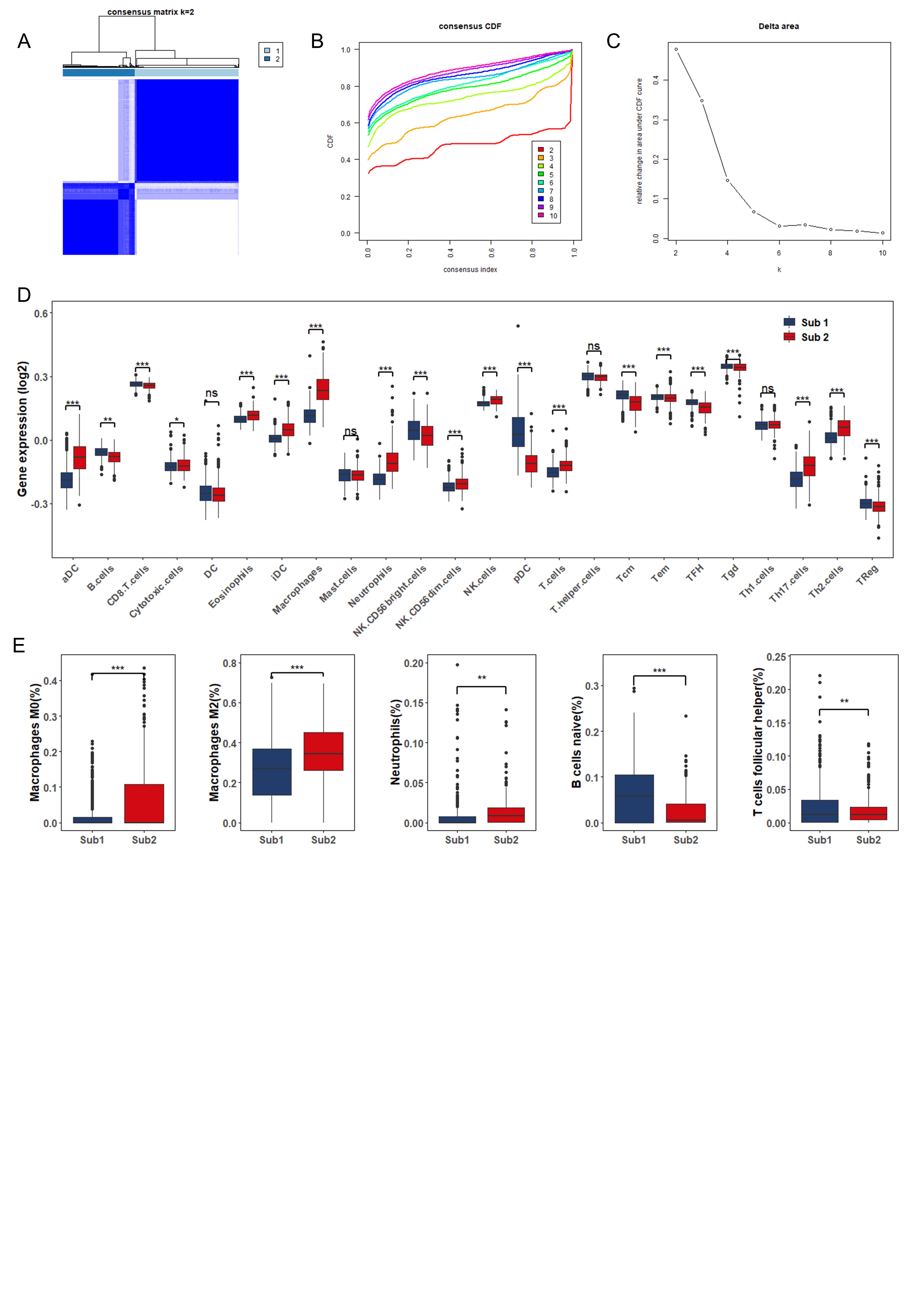


**Figure S1:** Glioma subtypes identified based on tumor-infiltrating immune cells: (A) Consensus clustering matrix for glioma samples when k = 2. (B) CDF plot showing a real random variable of its probability distribution based on consensus scores for each k (from 2 to 10, indicated by different colors). (C) Delta area curve. (D) Comparison of 24 types of tumor-infiltrating immune cells between Sub1 and Sub2 in TCGA database. (E) The infiltrating levels of the Macrophages M0, Macrophages M2, Neutrophils cells, naive B cells and follicular helper T cells in Sub1 and Sub2. **, P<0.01; ***, P<0.001;


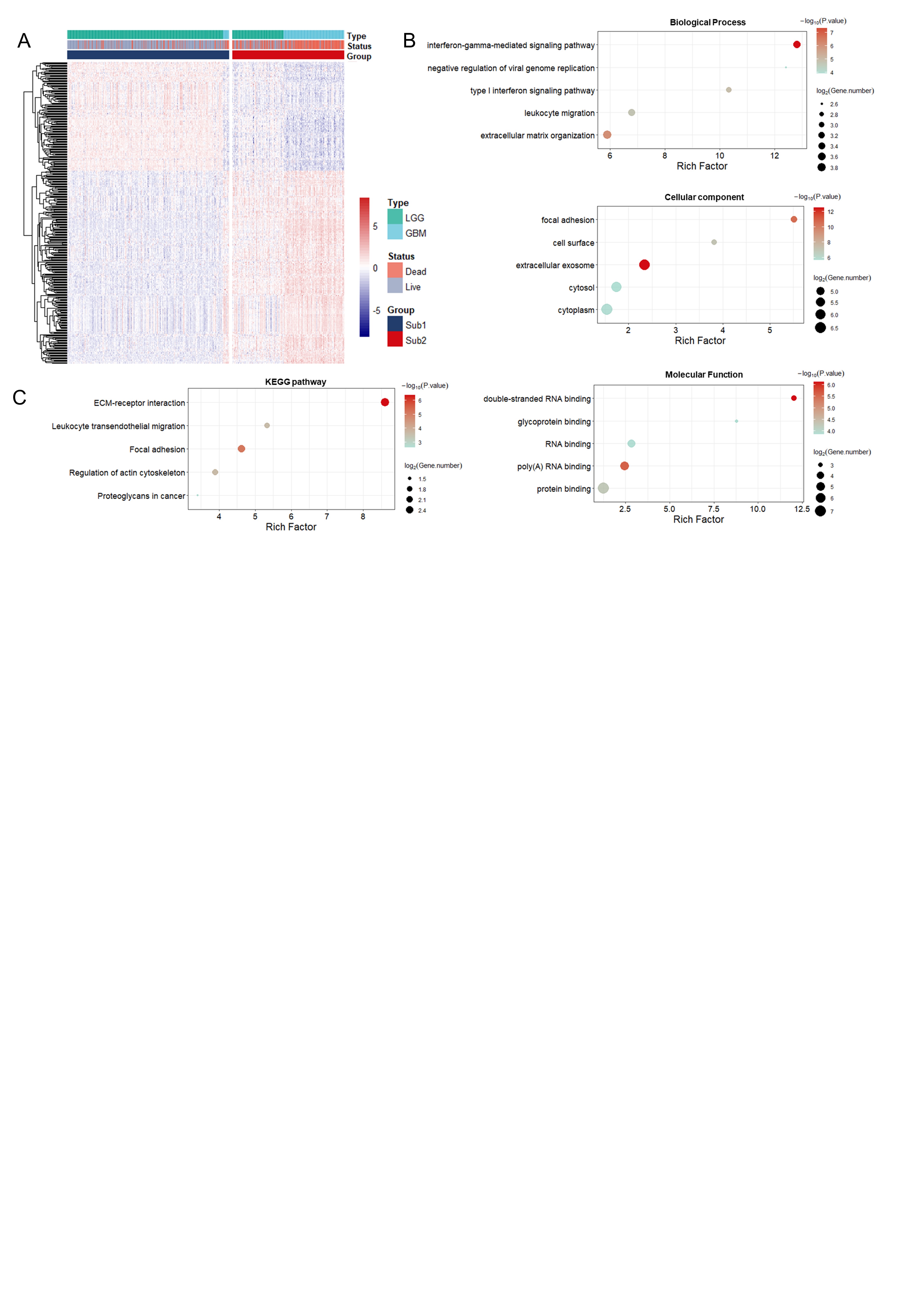


**Figure S2:** Identification and functional enrichment analysis of immune-associated RBPs in glioma patients: (A) Heatmap plot representing the differentially expressed immune-associated RBPs between Sub1 and Sub2 in glioma. (B) GO enrichment analysis of immune-associated RBPs based on Biological Processes (top), Cellular Component (middle) and Molecular Function (bottom). Top 5 enriched pathways were displayed on the figure. (C) Visualization of the top 5 enriched KEGG pathways by immune-associated RBPs.


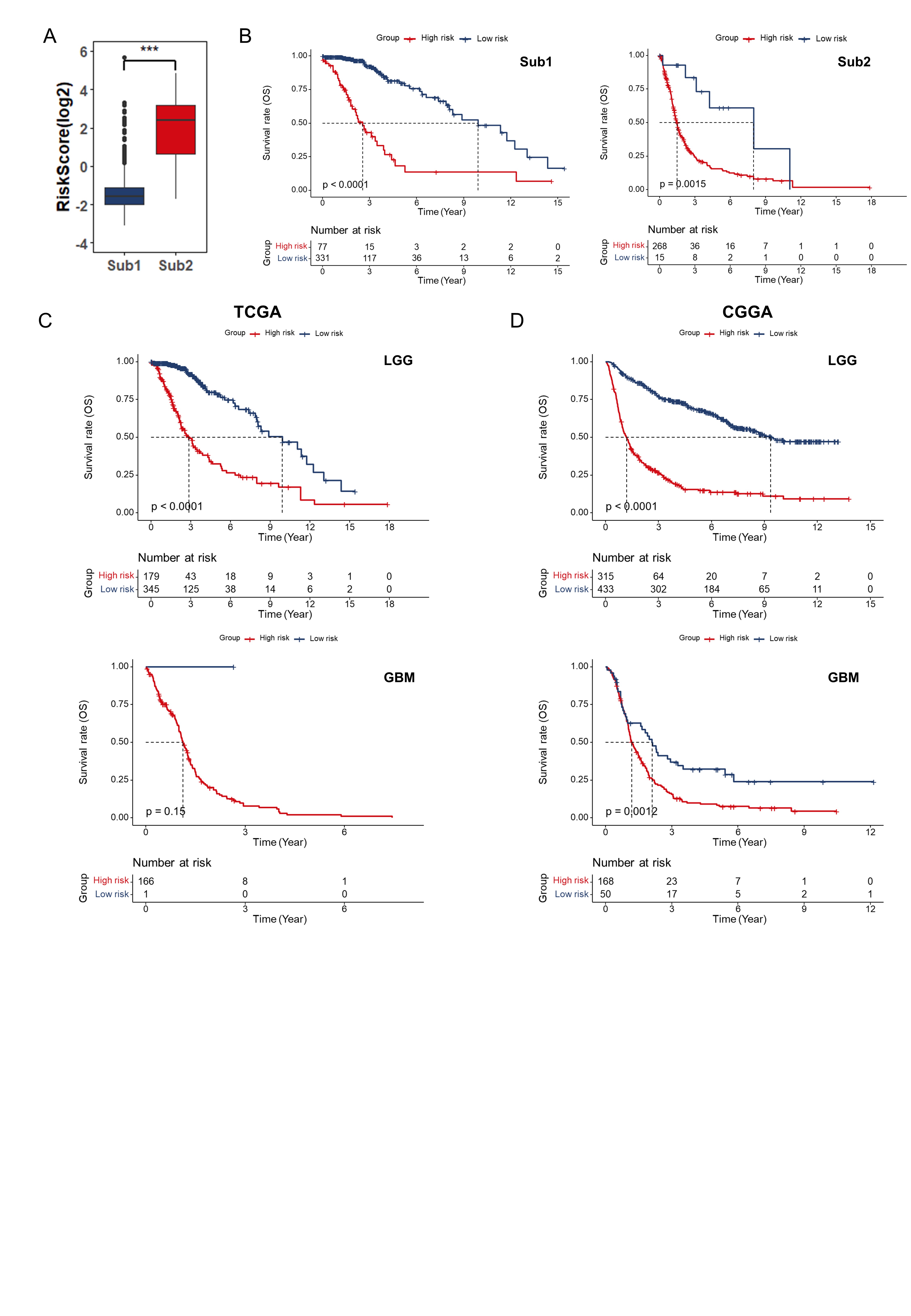


**Figure S3:** Identification and assessment of the 8 immune-associated RBPs prognostic signature for overall survival in glioma. (A) Distribution of risk scores in Sub1 and Sub2. (B) The prognosis values of immune-associated RBPs signature in Sub1 (left) and Sub2 (right) in TCGA database. (C) The prognosis values of immune-associated RBPs signature in LGG (top) and GBM (bottom) in TCGA database. (D) The prognosis values of immune-associated RBPs signature in LGG (top) and GBM (bottom) in CGGA database.
